# Association of placental mitochondrial DNA mutations on infant negative affectivity: modifying effects of maternal lifetime stress and infant sex

**DOI:** 10.1186/s13293-025-00717-4

**Published:** 2025-06-10

**Authors:** Agathe M. de Pins, Hsiao-Hsien Leon Hsu, Rosalind J. Wright, Kelly J. Brunst

**Affiliations:** 1 Icahn School of Medicine at Mount Sinai, New York, NY USA; 2 Department of Environmental Medicine, Icahn School of Medicine at Mount Sinai, New York, NY USA; 3 Institute for Exposomic Research, Icahn School of Medicine at Mount Sinai, New York, NY USA; 4 Department of Public Health, Icahn School of Medicine at Mount Sinai, New York, NY USA; 5 Department of Environmental and Public Health Sciences, University of Cincinnati, College of Medicine, Cincinnati, OH USA

**Keywords:** Placenta, Neurodevelopment, Mitochondria, Negative affectivity, Maternal stress

## Abstract

Neuropsychiatric and behavioral disorders impact over 15% of U.S. children, with sex differences in manifestation. Prenatal exposure to psychosocial stress predicts adverse neurodevelopmental outcomes, particularly during gestation. Mechanisms remain poorly understood. Research links prenatal stress exposures with placental mitochondrial DNA (mtDNA) mutational load, suggesting that disrupted mitochondrial placental function may play a role. We conceptualize that placental mitochondrial biomarkers reflect environmentally-induced oxidation that may contribute to mechanisms influencing neurodevelopment. Furthermore, as maternal stress can impact female and male children differently, this may in part explain sex differences in early childhood neurobehavioral outcomes. This study explores the association between placental mtDNA mutational load and negative affectivity in infants, and whether these associations are modified by maternal lifetime stress and fetal sex. Placenta samples (N = 394) were collected at delivery and whole mtDNA sequencing was performed to identify gene-specific mutational loads. Mothers completed the Infant Behavior Questionnaire-Revised (IBQ-R) when children were 6.69 ± 1.61 months of age and the Negative Affectivity factor was derived. Multivariable regression analyses were performed to model Negative Affectivity in relation to placental mtDNA mutational load, first adjusting for child sex and maternal age, self-reported race, and education. Lastly, we examined effect modification by maternal stress and fetal sex using cross-product terms and contrast statements. Results showed that higher mutational load in the MT_CYB region was positively associated with increased negative affectivity. Notably, interactions between mtDNA regions (MT_DLOOP and MT_ND), child sex, and maternal stress revealed that girls with higher mutational loads in these regions were at greater risk for increased negative affectivity, particularly under high maternal stress. These findings suggest that placental mtDNA mutational load could serve as a biomarker for neurodevelopmental risk, with sex-specific vulnerabilities influenced by maternal stress. This study underscores the importance of considering both environmental factors and sex differences in understanding early neurodevelopmental trajectories, and the potential of the placenta as a tool for early detection and intervention. Further research is needed to validate these findings and explore their implications for long-term child development.

**Highlights**

- Increased mutational load in the cytochrome B region of placental mtDNA is associated with higher infant negative affectivity.
- Girls exhibited greater sensitivity to mutations in the mitochondrial D-loop and NADH dehydrogenase regions, showing stronger links to negative affectivity compared to boys.
- Higher maternal lifetime stress amplified the impact of mitochondrial NADH dehydrogenase mutational load on negative affectivity in girls, highlighting gene-environment interactions.
- Findings underscore the placenta's role in integrating environmental and genetic factors that influence early temperament, and its potential role as a future biomarker.
- This is the first study connecting placental mitochondrial DNA mutations with infant temperament in a diverse population, revealing sex-specific and stress-modulated effects.

## Abstract (plain language summary)

The placenta is the main organ that connects a mother and her fetus, allowing them to communicate during pregnancy. Stressful experiences a mother faces before pregnancy, such as trauma and other stressors, can change how genes in the placenta function, influencing this communication. Mitochondria, the energy-producing parts of cells, play a critical role in these processes. Changes in mitochondrial gene activity can impact how the fetus develops. Our study focused on mitochondrial genes in the placenta that could affect the development of the fetal brain and therefore a child’s behavior. We also looked at whether these effects varied based on the child’s sex and the mother’s exposure to stress. We identified key mitochondrial genes that appear to influence a child’s behavior at six months of age. Additionally, we found that maternal stress had a stronger impact on gene activity in the placenta and brain development when the fetus was female compared to male. We identified which genes acted differently based on the child’s sex. This could allow us to better understand how maternal stress affects child development and identify specific biomarkers in the placenta that could predict a child’s risk for developmental or behavioral challenges later in life. In the future, this knowledge could help us create early interventions or treatments to support healthier outcomes for children.

## Background

Both animal and human studies suggest that cumulative maternal lifetime traumatic and non-traumatic stress-inducing experiences prior to conception influence offspring neurodevelopmental outcomes and temperament in early life that may have implications across the life course [1–5]. The association between cumulative maternal lifetime stress and child neurodevelopment is independent of stressors experienced during pregnancy [6–9]. Evidence also suggests that maternal lifetime stress impacts brain developmental differently depending on the child’s sex [10–12].

Temperament in infancy, including negative affectivity, is a harbinger of later neuropsychiatric disorders [13–15]. Cumulative stressors, including trauma experienced by women across their lifespan, is a well-documented factor contributing to neurobehavioral disorders in the next generation [28–30]. Yet, the biological mechanisms by which preconception adverse experiences are stored and communicated to impact future offspring neurodevelopmental outcomes remains poorly elucidated. The placenta plays an essential role in pregnancy and in leading to optimal fetal development and growth. Recent evidence from animal studies suggest that disrupted signaling at the maternal–fetal interface, specifically placental signaling, plays a central role in brain development [16, 17]. Molecular regulation of the placenta is also a major determinant of sex-specific developmental outcomes [18]. Moreover, optimal oxidant balance in the placenta is central to normal fetal brain development [19]. Mitochondria are major intracellular sources of reactive oxygen species [20]. The unique properties of mitochondrial DNA (mtDNA) enable the mitochondrial genome to integrate environmental cues, in the form of mtDNA heteroplasmies and/or copy number [21], and serve as a marker of oxidative stress [22, 23]. However, while mitochondrial physiology plays a crucial role in placental metabolism which is increasingly understood to impact fetal development, data from human studies remain sparse.

Our previous studies have linked maternal lifetime stressful experiences to adverse childhood neurobehavioral outcomes. In this context, it is important to also note that lifetime maternal stress can disrupt a range of physiological factors carried into pregnancy beyond placental mitochondrial processes that haves implications for fetal brain development. Cumulative lifetime stress is also linked to disruption of neuroendocrine hormones and immune factors [24–26] that influence fetal brain development in a sex-specific manner [27]. For example, our group has demonstrated associations between maternal childhood and lifetime trauma exposures with greater maternal hair cortisol levels over the course of pregnancy [24], as well as broader in utero hormone disruptions [26].

In order to begin to disentangle these complex associations, we set out to examine relationships between placental mtDNA mutation profiles on infant’s negative affectivity, a key temperament domain linked to later life neurobehavioral disruptions, as well as potential modifying effects of maternal lifetime stress and fetal sex on these associations. No prior study has examined associations between placental mtDNA mutational load as a dosimeter of maternal cumulative lifetime stress and child neurodevelopment, as proposed herein, as well as examining sex-specific effects.

## Methods

### Study population

The research involved individuals participating in the Programming of Intergenerational Stress Mechanisms (PRISM) study, a cohort centered on urban pregnancies. Its objective was to investigate the relationships between maternal psychosocial stress and various environmental factors like air pollution, smoking, and diet, and how they influence prenatal and postnatal development. Pregnant women, aged 18 and older, who spoke either English or Spanish, and were carrying a single fetus, were recruited. Exclusion criteria comprised maternal consumption of ≥7 alcoholic drinks per week before pregnancy, any alcohol intake after pregnancy detection, or being HIV+. Recruitment occurred at prenatal clinics in Boston (Beth Israel Deaconess Medical Center and East Boston Neighborhood Health Center) from 2011 to 2013, and at Mount Sinai Hospital in New York City from 2013 to 2018. At the time of analysis, 1,731 mothers were enrolled in the PRISM study. Of the enrolled participants, 936 completed the Infant Behavior Questionnaire-Revised (IBQR). Mitochondrial DNA mutational load was evaluated in a subset of placental samples obtained during delivery (N = 396) supported through supplemental funding. We additionally excluded two participants whose IBQ-R was completed outside the target age window (3–12 months), resulting in a final analytic sample of 394 infants. When comparing those included vs. those excluded in the analysis, there were no statistically significant differences based on maternal age, race, and education, child sex and exposure (lifetime maternal stress) and outcome (negative affectivity) scores. Among these 394 participants, complete data on maternal stress and infant temperament at 6 months of age were available for 361 infants. Birth details were gathered through postnatal questionnaires and medical chart reviews. Procedures were approved by the ethics committee at Icahn School of Medicine at Mount Sinai Institutional Review Board (IRB) (approval #12-00875A), and mothers provided written consent in their primary language (English or Spanish).

### Placental mtDNA sequencing/processing

Placenta samples were collected at delivery, biopsied on the fetal side (confirmed by 64 genotyping probes), processed utilizing RNAlater Stabilization Reagent (Quagen), and stored at -80 degrees celsius as previously described [32].

Placenta DNA was extracted using the Promega Wizard Genomic DNA Purification Kit (Promega – Madison, WI, USA) and mtDNA was amplified using long range PCR. DNA clean-up was performed using a Wizard SV Gel and PCR Clean-Up kit (Promega). After mtDNA amplification, approximately 100 ng of DNA was used as input for library preparation using the NEBNext Ultra II FS DNA Library Prep kit (NEB, Ipswich, MA). Quality and quantity of the library were confirmed using a bioanalyzer (Agilent, Santa Clara, CA) and NEBNext Library Quant Kit (NEB), respectively. Individually indexed and compatible libraries were then proportionally pooled and sequenced using a HiSeq 1000 sequencer (Illumina).

### Mitochondrial variant calling

Initial assessment of the mtDNA sequencing reads was conducted using FastQC software (https://www.bioinformatics.babraham.ac.uk/projects/fastqc/). We used BWA-MEM (v0.7.15) [33] for paired-end reads alignment to the revised Cambridge Reference Sequence (rCRS), GenBank NC_012920.1. We called variants according to the methods outlined previously [34] using the Genome Analysis Toolkit (GATK) Mutect2 variant caller [35] “mitochondrial mode”. Variants in the control region (CR) (positions 16,024–16,569 and 1–576), were realigned to a ChrM reference sequence shifted by 8000 base pairs. Coordinates were then converted back to their original positions and combined with variants from the non-control region into a merged VCF file. Variants marked with ‘fail’, ‘base-quality,’ ‘position,’ ‘blacklisted,’ and ‘strand’ flags in the VCF file’s filter column were excluded, as were deletions and insertions. Mitochondrial DNA contamination was estimated using Haplocheck (v1.3.3) [36], and haplogroups were determined using HaploGrep2 (v2.2.9) [37]. The variant allele fraction (VAF) was calculated as the ratio of alternate allele reads to total reads for a specific variant in a sample. Variants with a VAF between 0.05 and 0.98 were classified as heteroplasmic, while those with a VAF between 0.98 and 1.00 were considered homoplasmic. To minimize false positives, variants with alternative.

allele fractions below 5% were filtered out [34, 38]. Repeated sequencing was performed on 10% of the samples of batch 1, concordance in variant calls ranged between 80–100%.

### Infant negative affect

Mothers completed the Infant Behavior Questionnaire-Revised (IBQ-R) during a face-to-face visit when infants were 6.69 ± 1.61 months of age [39]. The IBQ-R is widely utilized for evaluating temperament in infants aged 3 to 12 months, across both English- and Spanish-speaking households. Mothers documented the frequency of 191 specific infant behaviors and reactions to real-life situations over the past 1 or 2 weeks. A trained research assistant presented each item to the mother and recorded her response, which was assessed on a 7-point Likert scale (ranging from 1 = never to 7 = always) on the questionnaire. We computed 14 temperament scales based on IBQ-R criteria, including four scales (Fear, Distress to Limitations/Frustration, Sadness, Falling Reactivity/Rate of Recovery) that have been identified to align with an overarching dimension of Negative Affectivity in factor analysis studies. In line with established scoring guidelines, we generated a composite score for global Negative Affectivity by averaging the mean scores of each of the four sub-scales. A higher score indicates a higher level of negative affectivity.

### Maternal lifetime stress exposure

Maternal lifetime exposure to traumatic and non-traumatic events was assessed via the 30-item Life Stressor Checklist-Revised (LSC-R). The LSCR includes events relevant to women (e.g. rape, abortion, interpersonal violence) and questions reflecting the participant’s appraisal of the severity of the negative impact using a Likert scale [ranging from 1 (not at all) to 5 (extremely)]. A weighted score of endorsed events that considered the negative impact of each was computed, with a range from 0 to 96. The LSCR has established test–retest reliability and validity in diverse populations [40, 41]. For purposes of this analysis, women were categorized as having high (LSCR weighted score > 9) or low stress (LSCR weighted score ≤ 9) based on median split.

### Covariates

Covariates included haplogroup, maternal education status (≤high school diploma), child sex, maternal age at delivery and LSCR-score (high or low). These covariates were chosen based on prior literature and biological relevance. Maternal education status was ascertained at enrollment. Child sex, maternal age at delivery and LSC-R score were obtained postnatally. Maternal mtDNA haplogroup was used as a proxy for genetic ancestry and categorized as African, Native American/Asian, or European.

### Statistical analysis

To examine the association between mitochondrial DNA (mtDNA) mutational load and infant Negative Affectivity, we utilized linear regression and interaction terms involving mtDNA mutational load at various mtDNA regions, child sex, and maternal stress (measured by LSC-R). Specifically, we evaluated the impact of mutational load across multiple mtDNA regions on Negative Affectivity by running individual linear regression models for each mtDNA region, including MT_CYB, MT_DLOOP, MT_ATP, MT_CO, MT_RNR, MT_tRNA, and MT_ND. Predictors were centered and scaled using their standard deviations to ensure comparability. Each model adjusted for child sex, maternal education level, maternal age at birth, and haplogroup. To explore potential effect modifications, we tested for interactions between mtDNA mutational load regions and child sex. Additionally, we assessed three-way interactions among mtDNA mutational load regions, child sex, and maternal lifetime stress (LSC-R). The continuous LSC-R score was dichotomized at its median (9) to form a binary variable representing high and low levels of maternal stress. For all models performed, we also checked for non-linearity in the relationships between predictors and the outcome variable. All analyses were performed using R (version 4.3.1, Vienna, Austria).

## Results

### Demographics

Demographic characteristics of the complete analytical sample (N = 394) stratified by child sex and maternal stress are presented in Table 1. Just under half (42.6%) of children were females. The average maternal age at birth was 29.4, with no significant differences between female and male children. The majority of women were from racial and ethnic minorities (51.3% from African haplogroup, 32.5% of Hispanic ethnicity, 27.6% from Native American or Indian haplogroup) with 37.4% of them reporting less than or equal to a high school education. Levels of maternal stress did vary by haplogroup and ethnicity (*p* < 0.05) but were not significantly different between child sex. Child sex did not vary based on haplogroup or ethnicity (*p* > 0.05). All individuals had complete mitochondrial mutational load data and had at least one heteroplasmy. Heteroplasmies were present in all mitochondrial genes. To facilitate comparisons across groups, we added *p*-values to Table 1 to show the significance of differences in sample characteristics. For continuous variables, we used t-tests when data were normally distributed (based on the Shapiro–Wilk test) and Wilcoxon rank-sum tests otherwise; for categorical variables, we used chi-square tests or Fisher’s exact tests, depending on data distribution and sample size.

**Table 1:** Cohort characteristics for all participants included in the analyses by child sex and high vs. low maternal lifetime stress

		Characteristics by child sex			By maternal stress		
	Total Sample (*N* = 396)	Female (*n* = 180)	Male (*n* = 226)	*p*-value	High Stress (*n* = *171*)	Low Stress (*n* = 192)	*p*-value
Child sex							
Female (%)	170 (43%)				68 (40)	90 (47)	0.2
Male (%)	226 (57%)				103 (60)	102 (53)	
Maternal Haplogroup							
African	204 (51.5%)	87 (21)	117 (21)	0.97	94 (55)	95 (49.5)	0.35
European	83 (21%)	35 (51)	48 (52)	0.87	31 (18)	47 (24.5)	0.18
Native American/Indian	109 (27.5%)	48 (28)	61 (27)	0.99	46 (27)	50 (26)	0.95
Maternal ethnicity							
Hispanic	126 (32.5%)	57 (34)	69 (31)	0.53	60 (35.3)	57 (30)	0.27
Maternal Education							
≤High School Diploma	148 (37.4%)	69 (41)	79 (35)	0.3	70 (41)	69 (36)	0.28
Mean maternal age at birth, years	29.4 (5.66)	29.4 (5.88)	29.3 (5.5)	0.95	28.7 (5.44)	29.8 (5.89)	0.1
High maternal lifetime stress (>median)	171 (47)	68 (18.7)	103 (28.4)	0.2			
Maternal lifetime stress (median, IQR)	9 (4–17)	9 (4–16)	10 (5–17)	0.1	18 (13–26)	4.5 (3–7)	–

Values are n (%) or mean ± SD

### Association between MT_CYB and negative affect

We conducted a series of linear regression analyses to examine the relationship between mitochondrial DNA (mtDNA) mutational load and infant Negative Affectivity outcomes, while considering the interactions with child sex and maternal lifetime stress (LSC-R).

**Fig. 1:**
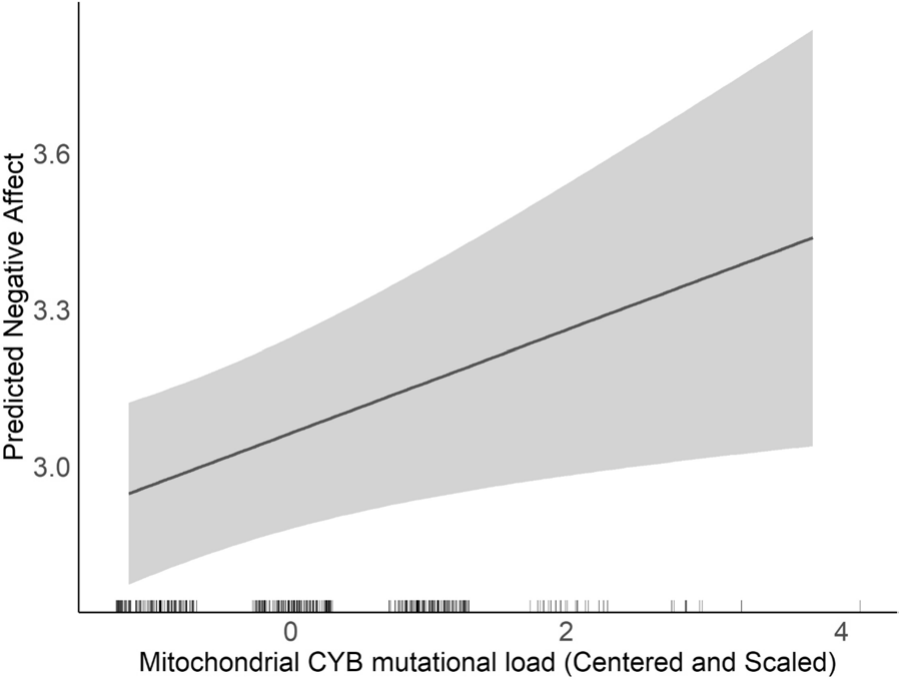
Association between mitochondrial CYB mutational load and predicted negative affect. This figure presents the results from a simple linear regression model, illustrating the predicted negative affect scores as a function of Mitochondrial CYB mutational load (β = 0.1, *p*-value = 0.01). The solid line represents the predicted values, and the shaded area indicates the 95% confidence interval. The model is adjusted for maternal education, child sex, haplogroup, and age at birth

All models were first adjusted for child sex, maternal education level, maternal age at birth, and haplogroup. As suggested by Fig. 1, we observed a significant positive association between MT_CYB mutational load and Negative Affectivity (β = 0.097, *p* = 0.015) suggesting that increased mutational load among the CYB region of mtDNA is associated with increased levels of infant negative affectivity. There were no other main effects of mtDNA mutational load on negative affectivity (all *p*-values > 0.05).

### Interaction between MT_DLOOP and child sex on negative affect

**Fig. 2:**
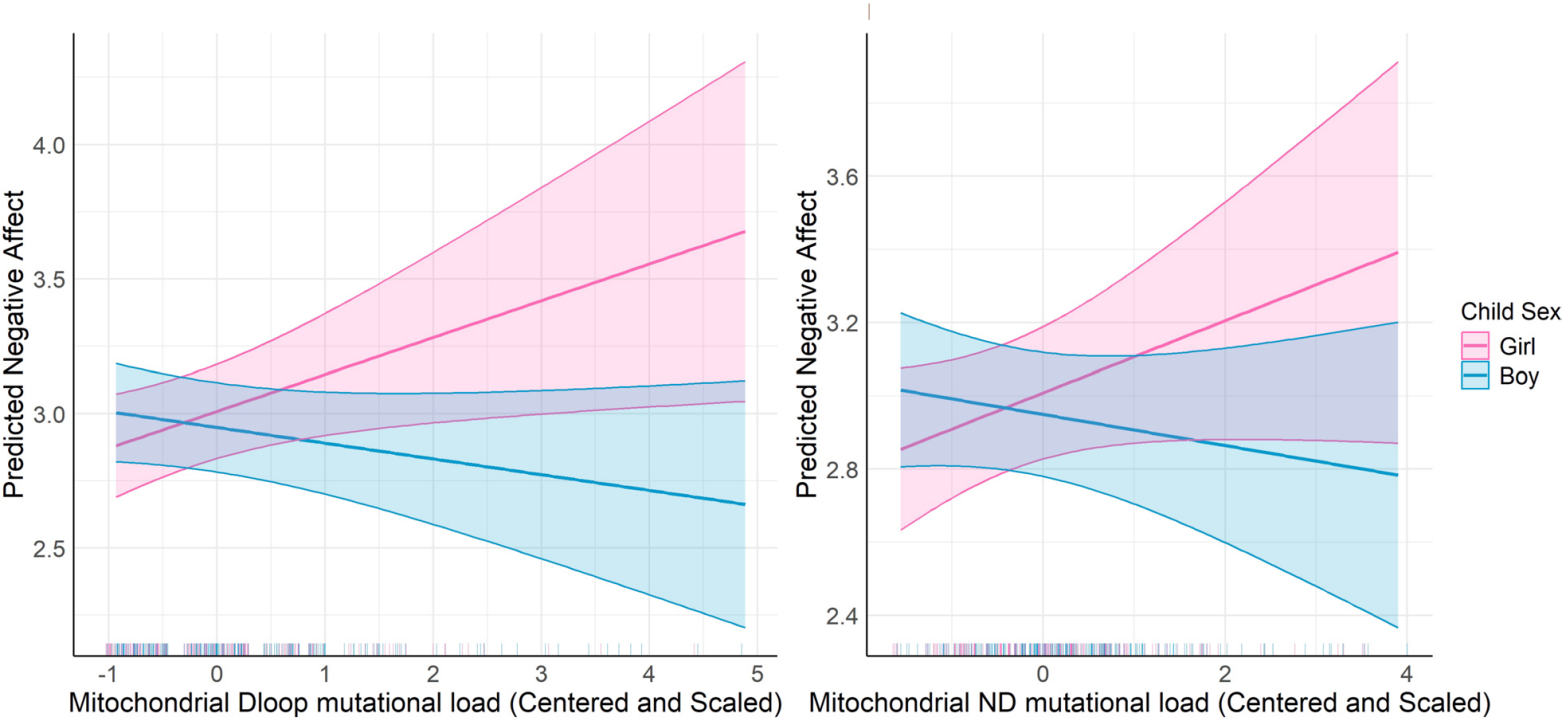
Interaction between Mitochondrial D-loop and ND Mutational Load and Child Sex on Predicted Negative Affect. The left panel shows the predicted negative affect scores in relation to Mitochondrial Dloop mutational load (β = 0.14, *p*-value = 0.02, Interaction Term β = −0.2, *p*-value = 0.01), while the right panel illustrates the predicted negative affect scores as a function of Mitochondrial ND mutational load (β = 0.1, *p*-value = 0.11, Interaction Term β = −0.13, *p*-value = 0.06). The interaction effects are assessed separately for boys (blue) and girls (pink), with shaded areas representing the 95% confidence intervals. The model is adjusted for maternal education, child sex, haplogroup, and age at birth

When looking at specific mutations, there was a significant interaction between MT_DLOOP mutational load and child sex (β = −0.194, *p* = 0.009) and a borderline significant interaction between MT_ND, mutational load and child sex (β = −0.135, *p* = 0.06), indicating that these relationships varied by child sex. Specifically, increased mutational load among the MT_DLOOP and MT_ND regions were associated with increased Negative Affectivity in girls compared to boys (Fig. 2). Sex-specific effects were not observed for other mtDNA mutational load regions and we did not observe any significant two-way interactions with LSCR.

### Three-way interaction among MT_ND, child sex, and LSC-R on negative affect

**Fig. 3:**
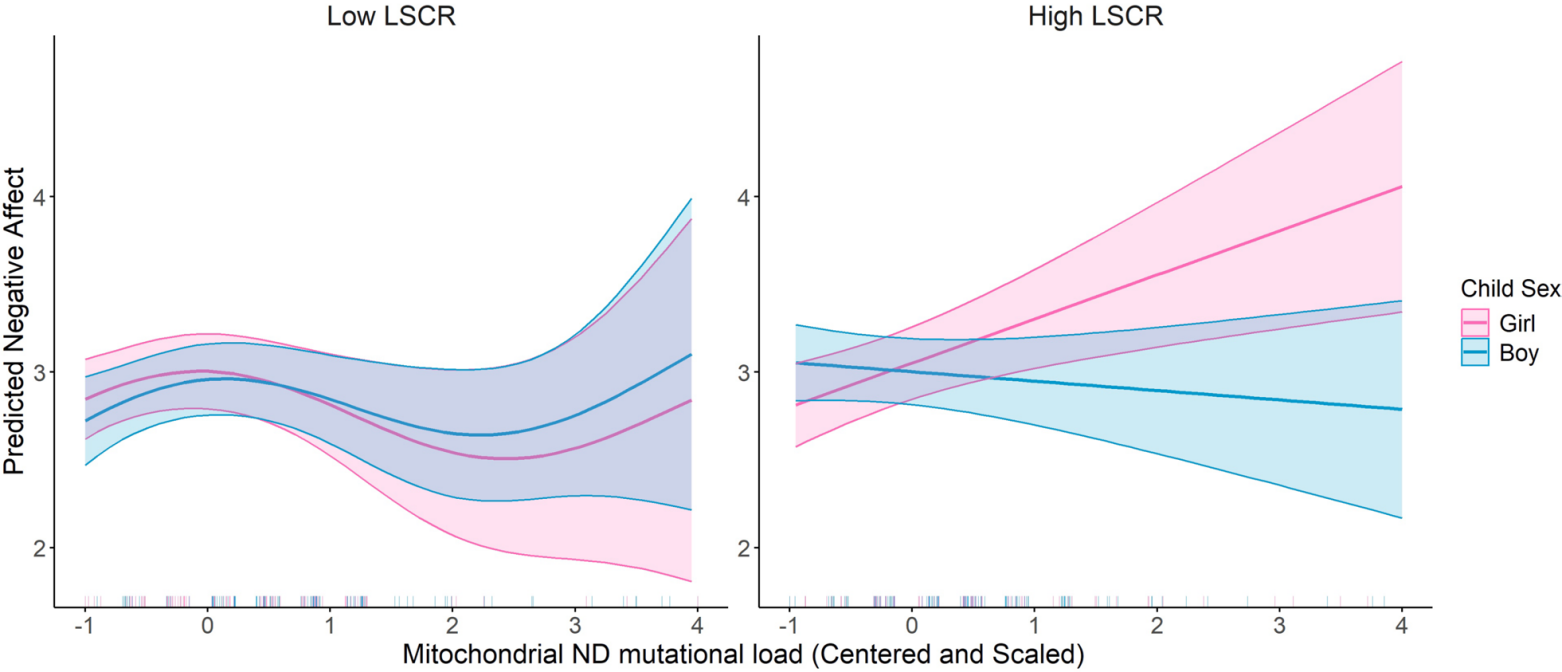
Three-way interaction effect of mitochondrial ND mutational load, child sex, and lifetime stress checklist (LSCR) on predicted negative affect. This figure demonstrates the predicted negative affect scores stratified by LSCR (low versus high, using a median cut-off). The interaction is further assessed by child sex, with predictions for boys (*blue*) and girls (*pink*). Shaded areas indicate the 95% confidence intervals. The model is adjusted for maternal education, child sex, haplogroup, and age at birth. The p-value for the smoothed Mitochondrial ND Mutational Load is 0.036 and the *p*-value for the smoothed interaction term between Mitochondrial ND Mutational Load, Child Sex, and LSCR is 0.005

We evaluated the three-way interaction between mtDNA mutational load regions, child sex, and maternal lifetime stress (LSC-R). This model included the main effects of mtDNA mutational load regions, child sex, LSC-R, maternal education level, maternal age at birth, and haplogroup. The results revealed a significant three-way interaction among MT_ND mutational load, child sex, and LSC-R (β = −0.307, *p* = 0.04). (Fig. 3) This indicates a complex interplay where the association between MT_ND mutational load and Negative Affectivity is modified by both child sex and maternal stress levels. The modifying effect of maternal stress on the relationship between MT_ND mutational load and Negative Affectivity was stronger in girls compared to boys suggesting that girls born to mothers who experience high levels of maternal stress that exhibit increased mutational load among the mt_ND region are more likely to experience elevated levels of Negative Affectivity.

## Discussion

This study identifies mtDNA regions whose mutational load is associated with increased negative affectivity in 6-month-old infants with girls being more affected than boys. It also suggests that maternal stress amplifies the impact of mtDNA mutation load on negative affectivity, especially in female infants. These findings suggest that placental mtDNA mutational load could serve as a biomarker for environmentally-induced neurodevelopmental risks, with sex-specific vulnerabilities.

Firstly, we identify that increased placental mutational load in the CYB region is positively associated with negative affectivity in 6-month olds. A recent study identified placental CYB gene expression in the second trimester as inversely associated with PM2.5 exposure [42], suggesting that this mitochondrial gene in the placenta might be particularly vulnerable to environmental stressors. The CYB mitochondrial gene codes for cytochrome B, part of complex III of the electron transport chain involved in oxidative phosphorylation to drive the production of ATP. It is the only gene is complex III that is encoded in the mitochondrial and not nuclear genome [43]. Poor function of cytochrome B can lead to cell death and increased oxidative stress in the placenta given the conversion of oxygen to ATP. Also, insufficient conversion of oxygen to ATP in the placenta, a high energy consuming organ, is likely to impact its function and subsequently impact neurodevelopment [44].

This study also identifies two mitochondrial DNA regions, the D loop and the ND genes, for which increases in mutational load influence negative affectivity in a sex-specific manner with increased mutational load leading to increased negative affectivity in girls but not boys. This suggests that the response of the placenta to higher mutational loads in these regions is different in girls and boys. Studies have shown that alterations to placental functioning can produce sex-specific transplacental signals to the developing brain [45]. The mitochondrial ND genes code for NADH dehydrogenase, which makes up complex I of the electron transport chain and is responsible for oxidizing NADH. NADH dehydrogenase is sensitive to cellular stress, with its function being disrupted within a hypoxic placenta. This has been shown to impact energy metabolism and fetal growth [46, 47]. Moreover, mutations in MT-ND6 and MT-ND5, both components of NADH dehydrogenase, have been associated with various psychiatric disorders [48, 49]. The D-loop is a non-coding region within the mtDNA that acts as a promoter for mtDNA genes. It is the most variable part of the mitochondrial genome and has a high mutation rate, with two hyper-variable regions [21, 50]. It helps the placenta adapt to hypoxic conditions by modulating energy production and plays a key role in ensuring adequate placental energy production during pregnancy [31, 51]. Similarly to the ND genes, mutations within the D-loop regions are also linked to pregnancy complications such as preeclampsia and intrauterine growth restriction [31, 52]. These hypoxic or improper conditions secondary to inadequate function of the electron transport chain could also potentially impair fetal neurodevelopment.

This study’s findings reinforce the idea of the placenta as a sexually dimorphic organ with sex specific effects. Studies have shown that there is a sex difference in responses to a placenta under hypoxic or stress-inducing conditions. In fact, one previous study found that higher mtDNA mutational load was associated with shorter gestational length, especially in females as well as lower birthweight in females compared to higher birthweight in males [31]. The placenta is made up of trophoblastic cells from the embryo that contain the embryo’s chromosomes, already creating a sexual character to the placenta. The placenta then generates hormones that will impact fetal development [53]. These conditions seem to impact different outcomes when it comes to male and female fetuses. In fact, male fetuses are at higher risk for early preterm birth and term preeclampsia whereas females have a higher incidence of intrauterine growth restriction, being small for gestational age and preterm preeclampsia [54–58]. Another study found that male-upregulated differentially expressed genes were enriched for mitochondrial transcripts [59, 60], which some have interpreted as a prioritization of fetal body growth in males while females may favor resilience [58]. These differences may also be driving the sex differences observed in this study when it comes to outcomes related to mtDNA and non-coding regions like the D-loop mutations impacting infant’s negative affectivity differently based on the infant’s sex.

Finally, this study highlights the importance of considering environmental factors such as stress when it comes to evaluating risk factors/biomarkers for neurodevelopment. In fact, child sex and maternal stress seem to be two key drivers of the relationship between mitochondrial mutational load and infant negative affectivity. We found that girls born to mothers experiencing high levels of stress and exhibit a high mutational load in the NADH dehydrogenase region are more likely to exhibit higher levels of negative affectivity than boys. These findings are consistent with literature in the field that suggests that girls have a greater vulnerability to prenatal maternal stress, with risks for anxiety and mood disorders, which are more consistent with negative affectivity in childhood [61, 62]. Boys on the other hand are more likely to exhibit externalizing behaviors and schizophrenia spectrum disorders [11]. These findings are also consistent with studies looking at environmental stressors in the form of air pollution, with exposure rendering boys more susceptible to autism spectrum disorder and impacting girls’ cognition [63–65].

This is the first study to our knowledge to examine associations between placental mtDNA mutational load and child neurodevelopment and whether associations vary by sex. This study represents an ethnically diverse sample of the population and highlights new ways to detect potential contributors to neuropsychiatric and behavioral deficits in children. Limitations of this study include a limited sample size of 394 infants which may impact the generalizability of the study to broader populations. This sample size also impacted our ability to look at single mutations to investigate their role in neurodevelopment.

Mitochondrial heteroplasmy reference levels in placental tissue were not available and so we did not adjust for cell-type heterogeneity, or characterize whether mutations were inherited or acquired.

Though we made a concerted effort to choose the most relevant confounding variables, other potentially relevant cofounders were not included such as maternal psychiatric history, pregnancy complications, or neighborhood. Furthermore, the IBQ-R relies on mother’s reporting and perception of their infant’s behavior which could be influenced by their cultural background and own stress levels. Also, the LSC-R questionnaire which relies on retrospective self-reporting could involve some recall bias. Nonetheless, both of these assessments well-known and validated tools for assessing infant behavior and lifetime stress, respectively. Finally, the study focuses on negative affectivity at 6 months of age which limits the ability to generalize those findings to broader neurobehavioral and neuropsychiatric deficits. However, early-life temperament has been shown to predict later life neurodevelopmental outcomes [61, 62]. We suggest that next steps should include examining the relationship between these genes and later life neurological behaviors.

## Conclusions

This study identifies new placental mitochondrial markers that possibly influence child’s neurodevelopment, sex differences in neural behavior, and more vulnerable to environmental influences. It highlights the potential of using the placenta as a marker of lifetime stress and possible future developmental issues in the child. It also shows the importance of creating accessible biomarkers for future prevention and the need for further research. 

## Data Availability

No datasets were generated or analysed during the current study.

## Abbreviations

mtDNA: Mitochondrial DNA
PM2.5: Fine particulate matter
IBQ-R: Infant behavior questionnaire revised
LSC-R: Life stressor checklist-revised
